# Magnitude of Dynamically Correlated Molecules as an Indicator for a Dynamical Crossover in Ionic Liquids

**DOI:** 10.1021/acs.jpcb.1c00653

**Published:** 2021-04-15

**Authors:** Małgorzata Musiał, Shinian Cheng, Zaneta Wojnarowska, Marian Paluch

**Affiliations:** Institute of Physics, University of Silesia in Katowice, Silesian Center for Education and Interdisciplinary Research, 75 Pulku Piechoty 1A, 41-500 Chorzow, Poland

## Abstract

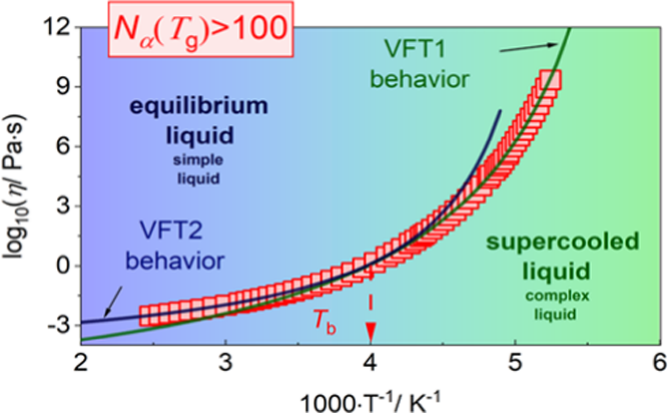

In this work, we show how the structure and intermolecular interactions affect the dynamic heterogeneity of aprotic ionic liquids. Using calorimetric data for 30 ionic samples, we examine the influence of the strength of van der Waals and Coulombic interactions on dynamic heterogeneity. We show that the dynamic length scale of spatially heterogeneous dynamics decreases significantly with decreasing intermolecular distances. Additionally, we assume that the magnitude of the number of dynamically correlated molecules at the liquid–glass transition temperature can be treated as an indicator for a dynamical crossover.

## Introduction

The concept of dynamic heterogeneity of supercooled liquids has been extensively studied^1−9^ since the work of Adam and Gibbs.^10^ Near the liquid–glass transition temperature, *T*_g_, the dynamics freezes drastically, while the structure of the system changes only slightly. Such abnormal molecular dynamics behavior is often rationalized in terms of correlated motions of the neighboring molecules. Adam and Gibbs introduced the idea of cooperatively rearrangement regions (CRRs), defined as a group of molecules that can change their configurations independently of their surroundings. The sizes of CRRs do not have to be constant in time and/or space, and the dynamics of molecules, which belong to different regions, are not identical.^11−14^ Consequently, molecules only a few nanometers away from each other may have relaxation rates that differ by several orders of magnitude. Hence, molecular dynamics is heterogeneous. These cooperating domains’ size increases as the temperature drops, which means that larger and larger groups of molecules in the supercooled liquid move cooperatively when approaching the glassy state. Therefore, the sizes of CRRs are often considered to play a crucial role in molecular dynamics near *T*_g_.

One of the most fundamental questions in this field is how the structure and intermolecular interactions affect the dynamic heterogeneity of supercooled liquids. It has been established that the number of dynamically correlated molecules, *N*_α_, at *T*_g_ is in the range of 100–400 for van der Waals liquids and H-bonded liquids, 400–600 for oxide glass formers and selenium, and 200–820 for polymers.^15−18^ The highest value (i.e., 818) was found for poly(vinyl chloride) by Capaccioli et al.^18^ Surprisingly, so far, there are no systematic studies of the dynamic heterogeneity of ionic systems. Recent research for protic ionic liquids (PILs) has shown that *N*_α_ at *T*_g_ for selected hydrochloride salts is smaller than that of their base counterparts.^19^ For example, the number of dynamically correlated molecules is equal to 225 for carvedilol base and *N*_α_ = 30 for carvedilol HCl. To explain these findings, the authors proposed that long-range electrostatic interactions induce a small scale of spatially heterogeneous dynamics in PILs. Simultaneously, the authors discovered that the relative change in PIL’s dynamic heterogeneity and its base depends on the molecular complexity of the salt cation. Hence, it cannot be ruled out that the topological constraints caused by simpler or more complex anions and cations result in a larger dynamic length scale of some ionic liquids (ILs). Moreover, since the above work was limited to protic ionic materials only, we still do not know whether the small scale of spatially heterogeneous dynamics is a general rule for all ion systems or just the unique feature of PILs. Thus, systematic studies are needed to draw a general conclusion about the magnitude of dynamic heterogeneity in ionic liquids.

The main aim of our work is to systematically study the dynamic heterogeneity in various aprotic ionic liquids. For this purpose, we will determine the number of dynamically correlated molecules at the liquid–glass transition temperature for aprotic ionic liquids (AILs) with different cation and anion structures and consequently with different strengths of electrostatic interactions as well as the various contributions of van der Waals forces and hydrogen bonds. This will allow us to provide general conclusions about the size of the dynamic heterogeneity of these materials. To determine the number of dynamically correlated molecules at *T*_g_, *N*_α_^D^(*T*_g_)_,_ the Donth method^20^ that requires only calorimetric data was applied.

We found that *N*_α_^D^(*T*_g_) decreases with the elongation of the carbon chain substituent in the cation or the anion. Thus, the decrease in intermolecular distances due to the stronger interactions with the increasing number of −CH_2_– groups induces a decrease in the dynamic length scale of spatially heterogeneous dynamics near the glass transition. At the same time, we noted that while maintaining the same strength of the van der Waals interaction (the same length of the alkyl chain), the obtained *N*_α_^D^(*T*_g_) decreases with the increase of the strength of the Coulomb interactions. Additionally, we investigated the evolution of log_10_ η(*T*^–1^) dependence and we observed the strong connection between the magnitude of the *N*_α_^D^(*T*_g_) value and existence/nonexistence of the intermediate temperature, *T*_b_ (identified with the onset of complex dynamics). Consequently, we assumed that the magnitude of *N*_α_^D^(*T*_g_) can be treated as an indicator for a dynamical crossover.

## Experimental Methods

### Materials

The supplier of the four ILs was Solvionic, whereas the rest was provided by IoLiTec. Note that in some cases, we have used the same batch of materials as in previous works. Full names, acronyms, and purities along with water contents (determined using the coulometric Karl Fischer method) of the tested ILs are listed in Table S1. The samples were dried and degassed under low pressure (1 kPa) at temperatures not exceeding 373 K.

### Viscosity Measurements

The viscosity was determined by means of an ARES G2 rheometer. In the vicinity of liquid–glass transition, aluminum parallel plates with a diameter of 8 mm were used. On the other hand, stainless steel geometries with diameters of 25 and 50 mm were used to obtain the viscosity in supercooled and normal liquid states, respectively. Oscillation shear experiments were performed in a frequency range from 0.05 to 100 rad·s^–1^ (7 points per decade) with the strain being dependent on temperature and changing from 0.1 to 1000%. The complex viscosity of the studied samples was determined directly from the frequency-independent part of the log_10 _η vs angular frequency graph. The relative uncertainty of the reported viscosity measurements *u*_r_(η) coming from calibration and temperature control did not exceed 7%.

### Differential Scanning Calorimetry (DSC)

Calorimetric experiments of the studied ILs were performed by means of Mettler Toledo DSC1STAR system equipped with a liquid nitrogen cooling accessory and an HSS8 ceramic sensor (a heat flux sensor with 120 thermocouples). Each sample with a mass of around 10–20 mg was measured in aluminum crucibles with a 40 μL volume. Prior to the measurement, the samples were annealed for 30 min at 373 K. Temperature ramps involved cooling to 143 K and then heating to 373 K with a rate of 10 K min^–1^. The samples were cycled at least three times to ensure reproducibility and high accuracy. During the experiments, the flow of nitrogen was maintained at a level of 60 mL min^–1^. Enthalpy and temperature calibrations were performed using indium and zinc standards.

## Results and Discussion

Herein, the number of dynamically correlated particles was calculated for 30 pure aprotic ionic liquids with different anion and cation structures and the length of the alkyl chain attached to the cation or anion. The full names of the tested materials along with their acronyms are listed in Table 1. The glass transition temperature of the examined ILs changes from 166.0 K for [C_4_C_1_pyr][DCA] to 230.8 K for [C_4_C_1_im][Cl]. Additionally, two binary mixtures and one ternary mixture were investigated (see Table 2). Due to this structural variety, interactions between cations and anions are significantly different in the selected materials.

**Table 1:** Acronyms of the Investigated Ionic Liquids along with the Glass Transition Temperature, *T*_g_, Intermediate Temperature, *T*_b_, Number of Dynamically Correlated Molecules, *N*_α_^D^(*T*_g_), Volume of ILs as the Sum of Ionic Volumes of the Constituting Cations, *v*_+_, and Anions, *v*_–_, and Volume of the Dynamically Correlated Molecules, *N*_α_^D^(*T*_g_)·(*v*_+_ + *v*_–_)

acronym	*T*_g_ (K)	*T*_b_ (K)	*N*_α_^D^(*T*_g_)	(*v*_+_ + *v*_–_)^a^ (nm^3^)	*N*_α_^D^(*T*_g_)·(*v*_+_ + *v*_–_) (nm^3^)
[C_4_C_1_im][Cl]	230.8	lack of viscosity data	120	0.245	29.4
[C_8_C_1_im][Cl]	220.1	lack of viscosity data	26.8	0.337	9.0
[C_4_C_1_im][OAc]	188.2	257.2	140	0.278	38.9
[C_2_C_1_im][DMP]	201.6	lack of viscosity data	112	0.252	28.2
[C_2_C_1_im][DEP]	203.8	not detected	69.8	0.302	21.1
[C_2_C_1_im][DBP]	201.1	not detected	20.6	0.402	8.3
[C_4_C_1_im][BF_4_]	188.2	not detected	78.3	0.276	21.6
[C_8_C_1_im][BF_4_]	193.8	not detected	36.7	0.368	13.5
[C_4_C_1_im][NO_3_]	188.2	257.9	111	0.260	28.9
[C_4_C_1_im][BETI]	183.0	not detected	49.8	0.504	25.1
[C_3_OC_1_pyr][FSI]	173.2	lack of viscosity data	120	0.254	30.5
[C_4_C_1_pyr][FSI]	167.4	not detected	57.5	0.361	20.7
[C_3_OC_1_pyr][TFSI]	188.4	not detected	60	0.329	19.7
[C_4_C_1_pyr][TFSI]	187.1	not detected	31.0	0.423	13.1
[C_4_C_1_pip][TFSI]	197.1	not detected	27.0	0.434	11.7
[C_4_C_1_aze][TFSI]	206.9	lack of viscosity data	30.0	0.445	13.4
[C_4_C_1_im][TFSI]	180.6	not detected	77.0	0.408	31.4
[C_8_C_1_im][TFSI]	189.5	not detected	57.1	0.500	28.6
[C_10_C_1_im][TFSI]	192.5	not detected	50.0	0.546	27.3
[C_2_C_1_im][TCM]	186.3	crystallization	221	0.232	51.3
[C_4_C_1_im][TCM]	190.3	251.3	199	0.278	55.3
[C_6_C_1_im][TCM]	193.8	267.4	154	0.324	49.9
[C_8_C_1_im][TCM]	194.5	279.3	102	0.360	43.9
[C_4_C_1_pyr][TCM]	173.3	241.5	176	0.293	51.5
[C_2_C_1_im][SCN]	177.0	208.9	168	0.235	39.5
[C_4_C_1_im][DCA]	176.8	250.1	187	0.267	49.9
[C_4_C_1_pyr][DCA]	166.0	230.8	124	0.282	34.9

a Calculated based on the data reported in refs (34−38).

**Table 2:** Acronyms, Glass Transition Temperatures, *T*_g_, Intermediate Temperatures, *T*_b_, and Numbers of Dynamically Correlated Molecules, *N*_α_^D^(*T*_g_) of the Investigated Mixtures

acronym	*T*_g_ (K)	*T*_b_ (K)	*N*_α_^D^(*T*_g_)
[C_4_C_1_im][TFSI] + [C_4_C_1_pyr][TFSI] (1:1 mol/mol)	189.4	not detected	59.3
[C_4_C_1_pyr][TFSI] + [C_4_C_1_im][TCM] (1:1 mol/mol)	186.8	251.9	140
[C_2_OC_1_pyr][TFSI] + [C_4_C_1_im][TCM] + [C_4_C_1_im][BF_4_] + (1:1:1)	183.1	244.3	120

One of the most widely used methods for calculating *N*_α_ has been described by Donth.^20,21^ Based on the fluctuation–dissipation theorem, this approach relates the entropy fluctuations to the specific heat capacity *c*_p_ recorded in the temperature range where the glass transition is observed. Donth et al. suggested that the width of the glass transition in *c*_p_(*T*) dependence is a good method to determine the size of temperature fluctuations.^22^ Consequently, the number of dynamically correlated particles, *N*_α_^D^(*T*_g_) can be calculated using the following relation
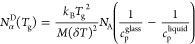
1where *M* is a molar mass of the tested sample, *T*_g_ denotes the glass transition temperature, *k*_B_ = 1.38 × 10^–23^ J·K^–1^ represents the Boltzman constant, *N*_A_ = 6.02 × 10^23^ mol^–1^ means the Avogadro constant, *c*_p_^glass^ and *c*_p_^liquid^ denote the isobaric heat capacities of glass and liquid at *T*_g_, respectively, and δ*T* is the average temperature fluctuation related to the dynamic glass transition (for a heating process with 10 K/min, *δT* = Δ*T*/2.5). An excellent description of the method with all details and explanations why the above procedure is suitable for the estimation of *N*_α_ at *T*_g_ is presented elsewhere.^19^

**Figure 1:**
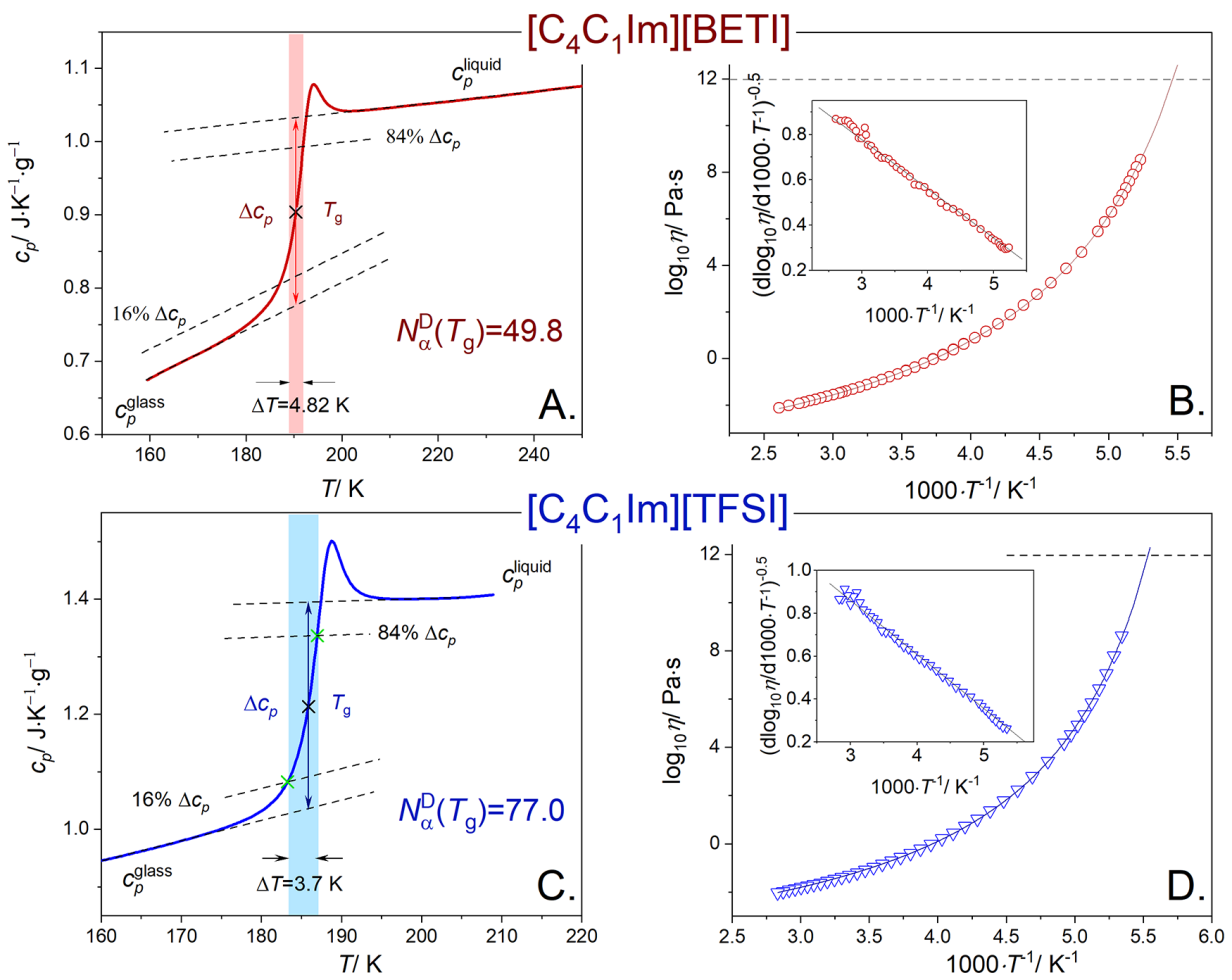
Scheme for the determination of the quantities needed for the calculation of *N*_α_^D^(*T*_g_) using the Donth method (eq 1) for the selected ILs: (A) [C_4_C_1_im][BETI] and (C) [C_4_C_1_im][TFSI]. The temperature dependence of viscosity for (B) [C_4_C_1_im][BETI] and (D) [C_4_C_1_im][TFSI]. The inset panel presents the result of Stickel analysis.

**Figure 2:**
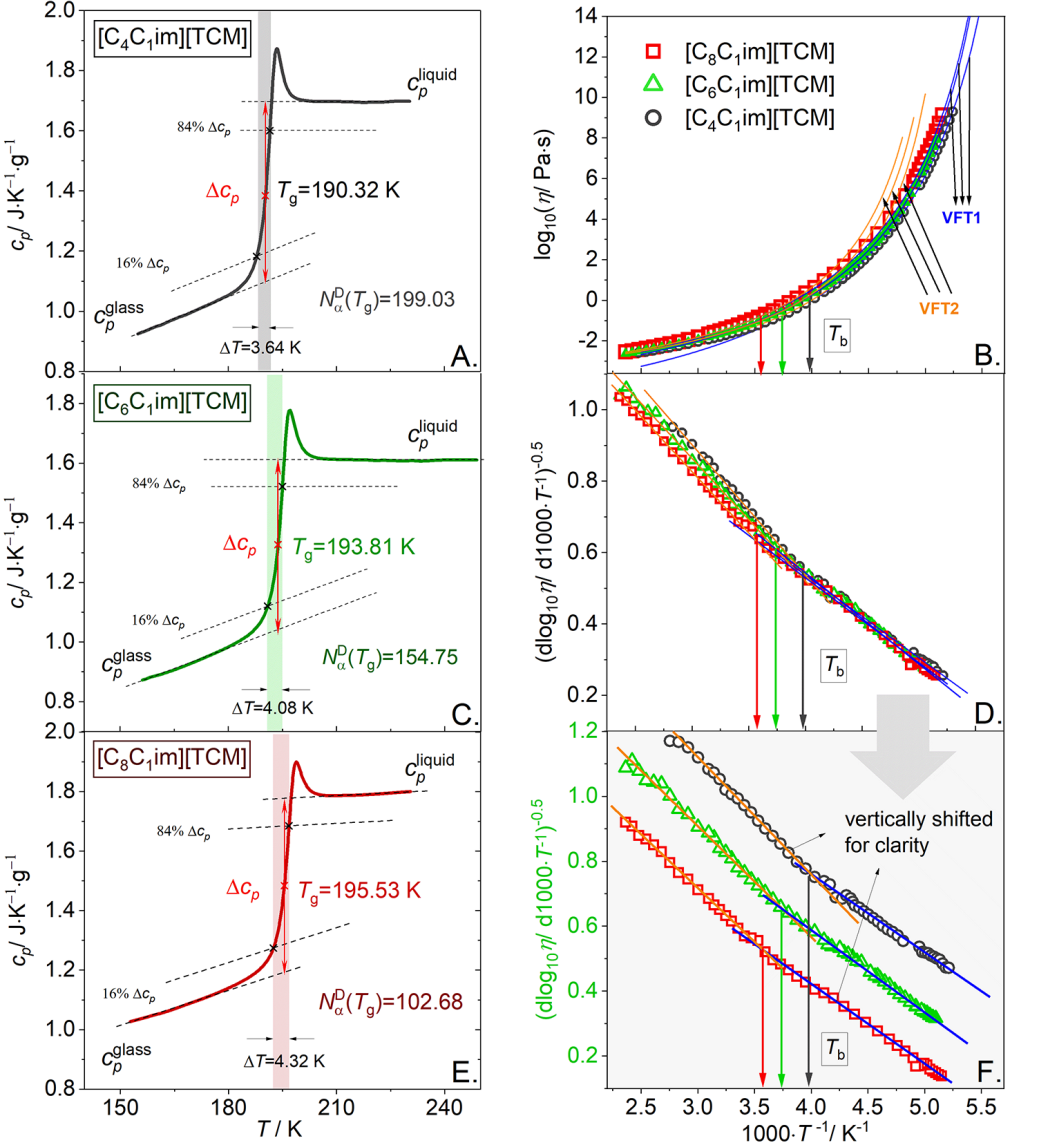
Scheme for the determination of the quantities used for the calculation of *N*_α_^D^(*T*_g_) using the Donth method (eq 1) for (A) [C_4_C_1_im][TCM], (C) [C_6_C_1_im][TCM], and (E) [C_8_C_1_im][TCM]. (B) The temperature dependence of viscosity for the [C_*n*_C_1_im][TCM] series. (D) The result of Stickel analysis for the [C_*n*_C_1_im][TCM] series. (F) The results of Stickel analysis for [C_*n*_C_1_im][TCM] shifted vertically for clarity.

Consequently, we determined *N*_α_^D^(*T*_g_) values using eq 1, and the results are summarized in Table 1. Additionally, for the selected ILs, the scheme for the determination of the quantities needed for the calculation of *N*_α_^D^(*T*_g_) using the Donth method is presented in Figures 1A,C and 2A,C,E. One can note from Table 1 that the highest *N*_α_^D^(*T*_g_) values are found for [C_2_C_1_im][TCM] and [C_4_C_1_im][TCM] having an anion with the symmetrical plane structure causing relatively weak cation–anion interactions due to equivalent chemical competition between same binding sites, i.e., between the three cyano groups (−CN groups). Moreover, it is almost impossible for the tricyanomethanide anion to form H-bonds with the cation.^23^ Consequently, ILs having an anion [TCM] are characterized by exceptionally low viscosity and high conductivity.^24^ Neves et al.^25^ reported a similar range of viscosity and conductivity for other ionic liquids with cyano-functionalized anions, i.e., thiocyanate-based and dicyanoamide-based ILs, and importantly, one can see that for all these ILs, *N*_α_^D^(*T*_g_) values are relatively large (see Table 1). More precisely, *N*_α_^D^(*T*_g_) for cyano-based ILs decreases in the order [TCM]^−^ > [DCA]^−^ > [SCN]^−^. This sequence is closely related to the change in the strength of the Coulomb interactions that occur at the bulk liquid. Namely, it is well known that an increase in ion size reduces the electrostatic attraction. Therefore, we can assume that the number of correlated molecules decreases with increasing electrostatic interaction strength, confirming the results obtained recently for protic ionic liquids.^19^ Another exciting finding coming from Table 1 is that in all abovementioned CN-based ILs, the change in Vogel–Fulcher–Tammann (VFT)^26−28^ dependence at *T*_b_ (interpreted as the intermediate temperature) is observed. According to literature data, *T*_b_ is identified with the onset of complex dynamics. There is a long list of evidence that some qualitative changes occur in the dynamics of glass-forming systems at *T*_b._(29) To demonstrate the change in dynamics occurring at a particular temperature above *T*_g_, Stickel et al.^30^ proposed a derivative analysis of temperature variations of the structural relaxation time ((d log_10_* x*/d1000/*T*)^−0.5^) to transforms VFT behavior into a linear dependence on inverse temperature. In this work, we analyzed the shear viscosity (η) determined by means of an ARES G2 rheometer and representative results together with Stickel analysis are presented in Figures 1B,D and 2B,D,F. Returning to ionic liquids with cyano groups, the obtained *T*_b_ is 71 ± 10 K higher than *T*_g_ in all cases except for [C_2_C_1_im][SCN], where the received *T*_b_ is only 31 K higher than the glass transition temperature.

**Figure 3:**
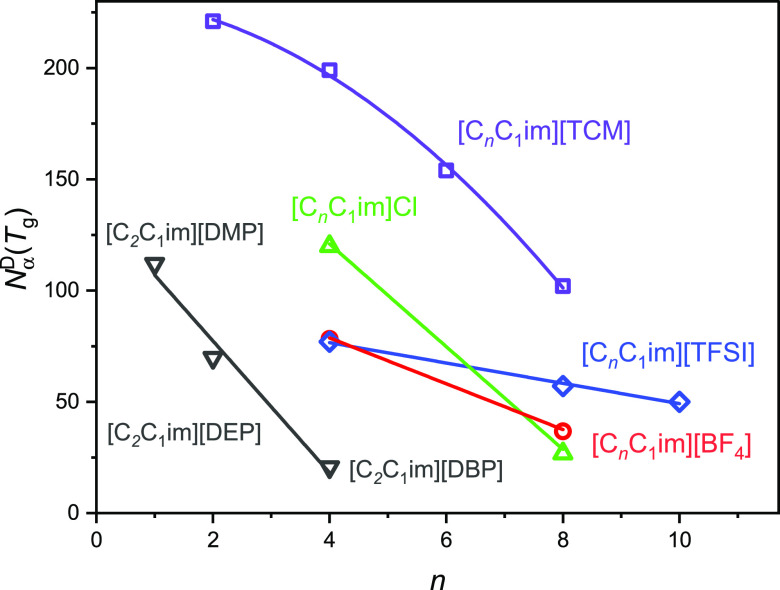
Number of correlated molecules as a function of alkyl chain length.

Additionally, from further inspection of the obtained results, it becomes apparent that the *N*_α_^D^(*T*_g_) value is strongly correlated with the alkyl chain length attached to the cation or anion. Namely, based on the results obtained for [C_*n*_C_1_im]Cl (*n* = 4, 8), [C_*n*_C_1_im][TFSI] (*n* = 4, 8, 10), [C_*n*_C_1_im][BF_4_] (*n* = 4, 8), [C_*n*_C_1_im][TCM] (*n* = 2, 4, 6, 8), and ([C_2_C_1_im][DMP], [C_2_C_1_im][DEP], [C_2_C_1_im][DBP]) series, it is obvious that *N*_α_^D^(*T*_g_) clearly decreases with the elongation of the alkyl chain length in the cation or anion (see Table 1 and Figure 3). Importantly, due to the lack of the H-bond network in [C_*n*_C_1_im][TCM], we may analyze the role of only two main interaction potentials in the ILs: electrostatic and nonelectrostatic (van der Waals). As reported by Vilas et al.,^31^ the electrostatic potential decreases initially until a stationary value at C_6_ (some authors suggested C_4_ instead of C_6_) is overlapped by the nearly linear van der Waals interaction functional (nonelectrostatic interaction potential), resulting in the highest overall interaction potential for [C_8_C_1_im][TCM]. Since at the same time, [C_8_C_1_im][TCM] has the lowest *N*_α_^D^(*T*_g_) value, we can assume that the decrease in intermolecular distances due to the stronger interactions with the increasing number of −CH_2_– groups induces the decrease in the dynamic length scale of spatially heterogeneous dynamics near the glass transition. In this context, it is worth noting that this statement agrees with the results reported by Koperwas et al. and Grzybowski et al. Namely, in the mentioned works, the authors observed that reducing the intermolecular distances as a result of compressing the system also causes its homogenization, i.e., a decrease in *N*_α_. It should be stressed that we do not observe visible changes in *N*_α_^D^(*T*_g_) values around *n* = 6 for [C_*n*_C_1_im][TCM] (*n* = 2, 4, 6, 8) and [C_*n*_C_1_im][NTf_2_] (*n* = 4, 8, 10) when, as was reported in the literature,^24,32,33^ the investigated ILs begin to form nanoscale aggregates (local microseparation leads to the formation of segregated polar and apolar domains in the bulk).

Another interesting finding from Table 1 is that *N*_α_^D^(*T*_g_) is almost independent of the size of the nonaromatic ring, i.e., nearly the same values were obtained for pyridinium-, piperidinium-, and azepanium-based ILs (*N*_α_^D^(*T*_g_) = 31, *N*_α_^D^(*T*_g_) = 27, and *N*_α_^D^(*T*_g_) = 30, respectively) with the same anion (TFSI) and the same alkyl chain length attached to the cation. It should be noted that these *N*_α_^D^(*T*_g_) values are one of the smallest among the investigated ILs, and *T*_b_ in all these cases was not detected.

**Figure 4:**
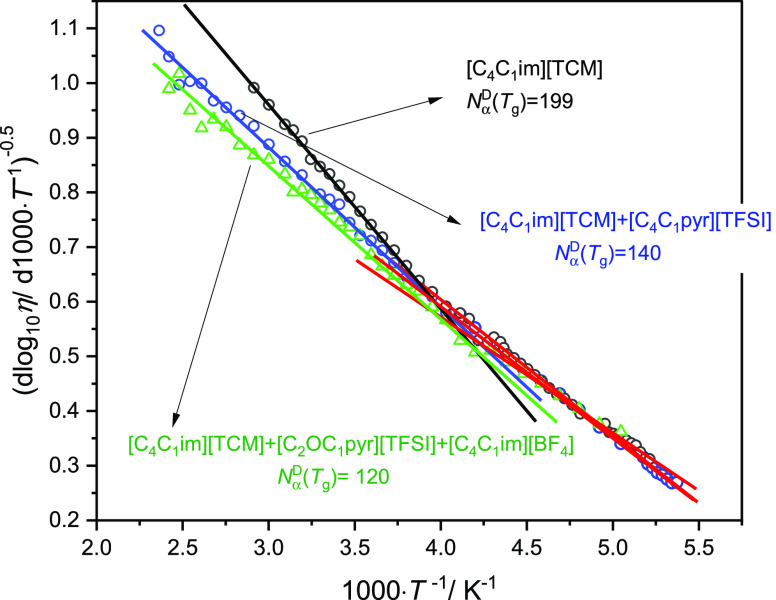
Result of Stickel analysis for pure [C_4_C_1_im][TCM] and binary and ternary mixtures of this IL.

Considering the above discussions, the next question arises: whether or not the change in VFT dependence at *T*_b_, characterizing a transition from simple dynamics to complex dynamics, is related to the number of dynamically correlated ions. To answer this question, we analyze the rest of the results. One can see in the insets of Figure 1B,D that for [C_4_C_1_im][BETI] and [C_4_C_1_im][TFSI] with *N*_α_^D^(*T*_g_) values equal to 49.8 and 77.0, respectively, a linear dependence in the whole investigated temperature range is observed, confirming a single-VFT-type behavior. Moreover, for other ILs where the determined *N*_α_^D^(*T*_g_) values are relatively small (<100), one VFT equation may be satisfactorily applied to describe the viscosity data in the whole range. On the other hand, if *N*_α_^*D*^(*T*_g_) is high enough (>100, which corresponds to the volume of the dynamically correlated molecules higher than ∼30 nm^3^—see Figure S1 in the Supporting information), we can detect the change from one VFT to another (see the representative results in Figure 2B,D,F). For the [C_*n*_C_1_im][TCM] (*n* = 4, 6, 8) series, *N*_α_^D^(*T*_g_) in each case is higher than 100, and in all cases, we observe a dynamic crossover. Notably, for nonionic glass formers (i.e., van der Waals liquids, polymers, and H-bonded systems) where *N*_α_^D^(*T*_g_) is higher than 100, a dynamic crossover is experimentally observed in these materials. Furthermore, the change from one VFT to another seems to be less visible with decreasing *N*_α_^D^(*T*_g_) value (see Figure 2A). To verify this observation, we chose [C_4_C_1_im][TCM] with the largest value of correlated molecules among the investigated ILs where we were able to measure viscosity in a broad thermodynamic range and at the same time with the most noticeable intersection. Consequently, we first mixed [C_4_C_1_im][TCM] with [C_4_C_1_im][TFSI] in a molar ratio of 1 to 1, and then we created a ternary mixture, namely, [C_2_OC_1_pyr][TFSI] + [C_4_C_1_im][TCM] + [C_4_C_1_im][BF_4_] (1:1:1). It turned out, first, that with increasing the components of the mixture, the parameter *N*_α_^D^(*T*_g_) in relation to [C_4_C_1_im][TCM] decreases and, second, the log_10_ η(*T*^–1^) dependence exhibits clear double VFT behavior for the binary mixture, and the crossover almost disappears entirely for the three-component mixture (see Figure 4).

## Conclusions

In summary, we obtained the number of dynamically correlated molecules using the Donth method for 30 ionic systems (27 pure ionic liquids and 3 mixtures). Among the investigated ILs, [C_2_C_1_im][TCM], [C_4_C_1_im][TCM], and [C_4_C_1_pyr][DCA] have the weakest cation–anion interactions and, at the same time, the highest number of correlated molecules at *T*_g_. We observed that the value of *N*_α_^D^(*T*_g_) clearly decreases with the elongation of the carbon chain substituent in the cation or anion. Thus, the decrease in intermolecular distances due to the stronger interactions with the increasing number of −CH_2_– groups causes a decrease in the dynamic length scale of spatially heterogeneous dynamics. Simultaneously, we noted that the obtained *N*_α_^D^(*T*_g_) decreases with the increase of the electrostatic interactions. Additionally, we observed the connection between the magnitude of the *N*_α_^D^(*T*_g_) value and the existence/nonexistence of the intermediate temperature *T*_b_ in the evolution of viscosity (log_10_ η(*T*^–1^)). This is especially interesting in the context of the physical meaning of *T*_b_, which is believed to be a border between complex (*T* < *T*_b_) and simple (*T* > *T*_b_) dynamics. Thus, if the number of correlated molecules is big enough (*N*_α_^D^(*T*_g_) > 100, which corresponds to the volume occupied by ionic species equal to ∼30 nm^3^), we can detect that change from simple to complex dynamics, i.e., two VFT equations with two different sets of fitting parameters are necessary to describe the temperature dependence of log_10_ η. Consequently, we assumed that the magnitude of *N*_α_^D^(*T*_g_) can be treated as an indicator for a dynamical crossover.

## Supporting Information Available

Full names, acronyms, suppliers, purities, and water contents of the examined ILs (Table S1) and volume of the dynamically correlated molecules as a function of alkyl chain length and number of dynamically correlated molecules (Figure S1) (PDF)
