# Supplementary material for: Magnitude of Dynamically Correlated Molecules as an Indicator for a Dynamical Crossover in Ionic Liquids
Source: J Phys Chem B. 2021 Apr 15;125(16):4141–7. doi: 10.1021/acs.jpcb.1c00653 (PMC8154596; doi:10.1021/acs.jpcb.1c00653)
Supplement: Supplementary file 1 — jp1c00653_si_001.pdf [file jp1c00653_si_001.pdf]

SUPPORTING INFORMATION

The Magnitude of Dynamically Correlated Molecules as an Indicator for a Dynamical Crossover in Ionic Liquids

Małgorzata Musiał*, Shinian Cheng, Zaneta Wojnarowska, Marian Paluch

*Corresponding Author: malgorzata.musial@smcebi.edu.pl

Table S1. Full name of investigated ionic liquids along with acronyms, suppliers, purities, and water contents of the examined ILs.

Ionic liquid	Acronym	Supplier	Purity[a]/ %;	Water content[b]/ ppm
1-butyl-3-methylimidazolium chloride	$[C_4C_1im][Cl]$	Iolitec	>98	890
1-methyl-3-octylimidazolium chloride	$[C_8C_1im][Cl]$	Iolitec	>99	1420
1-butyl-3-methylimidazolium acetate	$[C_4C_1im][OAc]$	Iolitec	>98	1350
1-ethyl-3-methylimidazolium dimethyl phosphate	$[C_2C_1im][DMP]$	Iolitec	>98	110
1-ethyl-3-methylimidazolium diethyl phosphate	$[C_2C_1im][DEP]$	Iolitec	>98	570
1-ethyl-3-methylimidazolium dibutyl phosphate	$[C_2C_1im][DBP]$	Iolitec	>97	620
1-butyl-3-methylimidazolium tetrafluoroborate	$[C_4C_1im][BF_4]$	Iolitec	>99	280
1-methyl-3-octylimidazolium tetrafluoroborate	$[C_8C_1im][BF_4]$	Iolitec	>99	220
1-butyl-3-methylimidazolium	$[C_4C_1im][NO_3]$	Iolitec	>98	320

nitrate				
1-butyl-3-methylimidazolium bis(perfluoroethylsulfonyl)imide	$[C_4C_1im][BETI]$	Iolitec	>99	<100
1-(3-methoxypropyl)-1-methylpyrrolidinium bis(fluorosulfonyl)imide	$[C_3OC_1pyr][FSI]$	Solvionic	99.9	<100
1-butyl-1-methylpyrrolidinium bis(fluorosulfonyl)imide	$[C_4C_1pyr][FSI]$	Solvionic	99.9	<100
1-(3-methoxypropyl)-1-methylpyrrolidinium bis((trifluoromethyl)sulfonyl)amide	$[C_3OC_1pyr][TFSI]$	Solvionic	99.9	<100
1-butyl-1-methylpyrrolidinium bis(trifluoromethylsulfonyl)imide	$[C_4C_1pyr][TFSI]$	Solvionic	99.9	<100
1-butyl-1-methylpiperidinium bis(trifluoromethylsulfonyl)imide	$[C_4C_1pip][TFSI]$	Iolitec	>99	<100
1-butyl-3-methylimidazolium bis(trifluoromethylsulfonyl)imide	$[C_4C_1im][TFSI]$	Iolitec	>99	<100
1-methyl-3-octylimidazolium bis(trifluoromethylsulfonyl)imide	$[C_8C_1im][TFSI]$	Iolitec	>98	240
1-decyl-3-methylimidazolium bis(trifluoromethylsulfonyl)imide	$[C_{10}C_1im][TFSI]$	Iolitec	>99	280
1-ethyl-3-methylimidazolium tricyanomethanide	$[C_2C_1im][TCM]$	Iolitec	>98	1190[c]
1-butyl-3-methylimidazolium tricyanomethanide	$[C_4C_1im][TCM]$	Iolitec	>98	990[c]
1-hexyl-3-methylimidazolium tricyanomethanide	$[C_6C_1im][TCM]$	Iolitec	>98	1232[c]
1-methyl-3-octylimidazolium tricyanomethanide	$[C_8C_1im][TCM]$	Iolitec	>98	1457[c]
1-butyl-1-methylpyrrolidinium tricyanomethanide	$[C_4C_1pyr][TCM]$	Iolitec	>98	520

1-ethyl-3-methylimidazolium thiocyanate	$[C_2C_1im][SCN]$	Iolitec	> 98	1750
1-butyl-3-methylimidazolium dicyanoamide	$[C_4C_1im][DCA]$	Iolitec	>98	310
1-butyl-1-methylpyrrolidinium dicyanamide	$[C_4C_1pyr][DCA]$	Iolitec	>98	250

[a] declared by Supplier; [b] determined using the coulometric Karl Fischer method; [c] reported in ref. S1

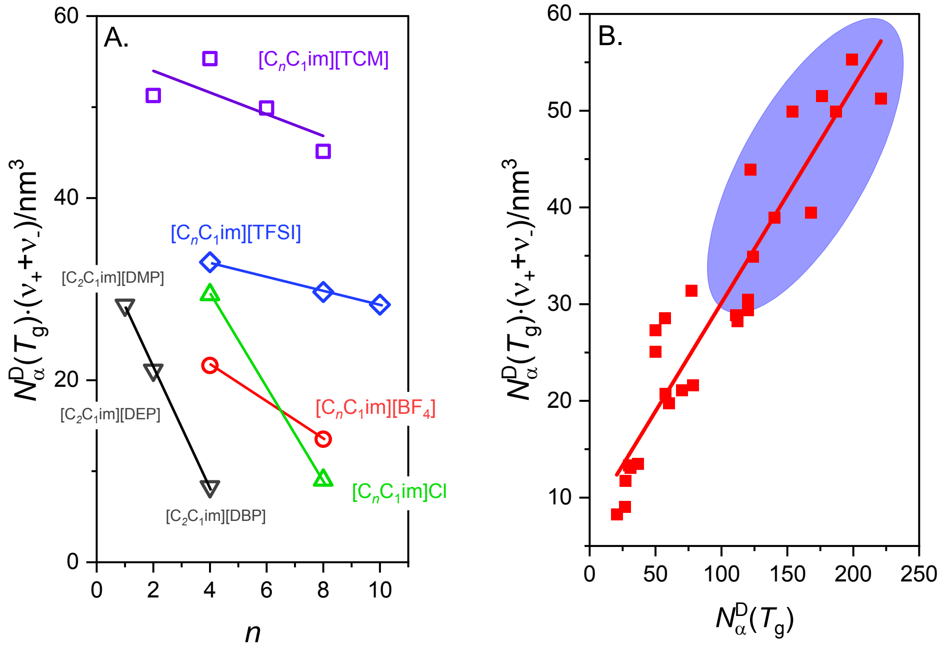

Figure S1. The volume of the dynamically correlated molecules: A. as a function of alkyl chain length; B. as a function of $N_\alpha^D(T_g)$.

Reference

S1. Musiał, M.; Cheng, S.; Wojnarowska, Z.; Paluch, M. Density, viscosity, and high-pressure conductivity studies of tricyanomethanide-based ionic liquids. *J. Mol. Liq.* **2020**, *317*, 113971.
